# Role of hydrogen sulfide in health and disease

**DOI:** 10.1002/mco2.661

**Published:** 2024-08-16

**Authors:** Yu‐Qing Jin, Hang Yuan, Ya‐Fang Liu, Yi‐Wen Zhu, Yan Wang, Xiao‐Yi Liang, Wei Gao, Zhi‐Guang Ren, Xin‐Ying Ji, Dong‐Dong Wu

**Affiliations:** ^1^ Henan International Joint Laboratory for Nuclear Protein Regulation School of Basic Medical Sciences, School of Stomatology Henan University Kaifeng Henan China; ^2^ School of Clinical Medicine Henan University Kaifeng Henan China; ^3^ Faculty of Basic Medical Subjects Shu‐Qing Medical College of Zhengzhou Zhengzhou Henan China; ^4^ School of Stomatology Henan University Kaifeng Henan China; ^5^ Department of Stomatology Huaihe Hospital of Henan University Kaifeng Henan China

**Keywords:** antioxidant, apoptosis, cancer, hydrogen sulfide, inflammation

## Abstract

In the past, hydrogen sulfide (H_2_S) was recognized as a toxic and dangerous gas; in recent years, with increased research, we have discovered that H_2_S can act as an endogenous regulatory transmitter. In mammals, H_2_S‐catalyzing enzymes, such as cystathionine‐β‐synthase, cystathionine‐γ‐lyase, and 3‐mercaptopyruvate sulfurtransferase, are differentially expressed in a variety of tissues and affect a variety of biological functions, such as transcriptional and posttranslational modification of genes, activation of signaling pathways in the cell, and metabolic processes in tissues, by producing H_2_S. Various preclinical studies have shown that H_2_S affects physiological and pathological processes in the body. However, a detailed systematic summary of these roles in health and disease is lacking. Therefore, this review provides a thorough overview of the physiological roles of H_2_S in different systems and the diseases associated with disorders of H_2_S metabolism, such as ischemia–reperfusion injury, hypertension, neurodegenerative diseases, inflammatory bowel disease, and cancer. Meanwhile, this paper also introduces H_2_S donors and novel release modes, as well as the latest preclinical experimental results, aiming to provide researchers with new ideas to discover new diagnostic targets and therapeutic options.

## Graphical abstract

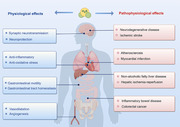

## 1 INTRODUCTION

Over the years, hydrogen sulfide (H_2_S) has been known for its rotten egg‐like odor, toxicity, and environmental hazard. The toxicity of H_2_S is mainly due to the inhibition of cytochrome *c* oxidase (COX) in mitochondria, leading to chemical asphyxia of cells.^1^, ^2^ COX is a vital electron transmitter in the respiratory chain, which participates in cellular respiration. Its activity is inhibited, which reduces oxygen utilization in mitochondria, leading to cell hypoxia.^3^, ^4^ In recent years, human understanding of H_2_S has gradually shifted from toxic substances to gas transmitters with therapeutic drug potential. In 1989, H_2_S was proven to exist in the human brain and may play a certain physiological role.^5^ In 1996, Japanese scientists demonstrated that H_2_S is a potential signaling molecule that can be produced by cystathionine‐β‐synthase (CBS) and is involved in neurotransmission.^6^ The following year, they discovered that cystathionine‐γ‐lyase (CSE) is another enzyme that produces.^7^ Subsequently, Wang et al.^8^ confirmed that H_2_S is the third physiological signaling molecule, except for carbon monoxide (CO) and nitric oxide (NO). Since then, the field of sulfide research has developed rapidly, and the research results have become richer; in a 2005 paper, Blackstone et al.^9^ reported in a pioneering manner that H_2_S can induce a reversible pseudo‐death‐like state in mice. They hypothesized that H_2_S‐mediated induction of pseudo‐death may have beneficial medical applications, such as ischemia–reperfusion injury (IRI) or organ preservation after trauma.^9^

In addition to the cytoprotective effects shown in IRI, H_2_S can act as an endogenous regulatory transmitter in other physiological states, including vasodilation, neuronal activity, gastrointestinal motility, and so forth.^10^, ^11^, ^12^, ^13^, ^14^, ^15^ A few pathologies are also strongly associated with H_2_S, such as the IRIs mentioned above, neurodegenerative diseases (ND), pain, inflammatory bowel disease (IBD), and cancer.^16^, ^17^, ^18^, ^19^ For exogenous H_2_S therapy, the manner of H_2_S delivery is important. At present, the main methods of H_2_S delivery include direct inhalation and H_2_S donor. Yet, all these delivery methods have certain limitations. Although recent studies have identified many small molecule H_2_S donors, they still perform poorly regarding stability, solubility, targeting, and half‐life. Nowadays, the main way to constitute an H_2_S delivery system is to combine H_2_S donors with polymers by covalent linkage and physical entrapment. The delivery systems optimize the therapeutic efficacy with higher stability and bioavailability compared with small molecule H_2_S donors.

In this article, we will focus on the role of H_2_S in human health and disease. The multiple physiological and pathophysiologic functions of H_2_S will be discussed systemically and comprehensively. We will delve into the novel H_2_S donors in anticipation of expanding new therapeutic ideas for researchers.

## 2 PHYSICOCHEMICAL PROPERTIES, PRODUCTION, AND METABOLISM OF H_2_S

### 2.1 General physicochemical properties of H_2_S

H_2_S is a colorless and highly toxic flammable gas with a unique rotten egg or sewage odor. Its molecular weight is 34.08, and its vapor density is heavier than air, making it easier to diffuse at lower points.^10^, ^20^ As a binary weak acid, hydrosulfuric acid is an aqueous solution of H_2_S that can reach dissociation equilibrium at room temperature (25°C). The solution concentration in a saturated state is 0.11 mol dm^−3^, and its pH value is approximately 4.0. At 37°C and pH 7.4, p*K*_α1 _= 6.76, there is about 20% H_2_S, 80% HS ^−^ and a small amount of negligible S^2‐^ in extracellular fluid.^21^ At the same time, H_2_S is also a small gas molecule with high lipophilicity, which allows it to freely pass through the lipid bilayer structure of the cell membrane.^22^ H_2_S is a compound similar to water molecules that can be oxidized into sulfur dioxide, sulfate, thiosulfate, and elemental sulfur. In the body, H_2_S can be oxidized to sulfates and thiosulfates, excreted in the urine. Some studies suggest that urinary thiosulfates can serve as one of the biomarkers of H_2_S poisoning.^20^, ^23^

### 2.2 Production of endogenous H_2_S

In mammalian cells, enzyme catalysis and nonenzyme catalysis are two ways to produce endogenous H_2_S. Some studies have shown that enzyme catalysis is the main production route, and CBS, CSE, and 3‐mercaptopyruvate sulfurtransferase (3‐MST) are the three key enzymes of this route.^24^, ^25^, ^26^

**FIGURE 1:**
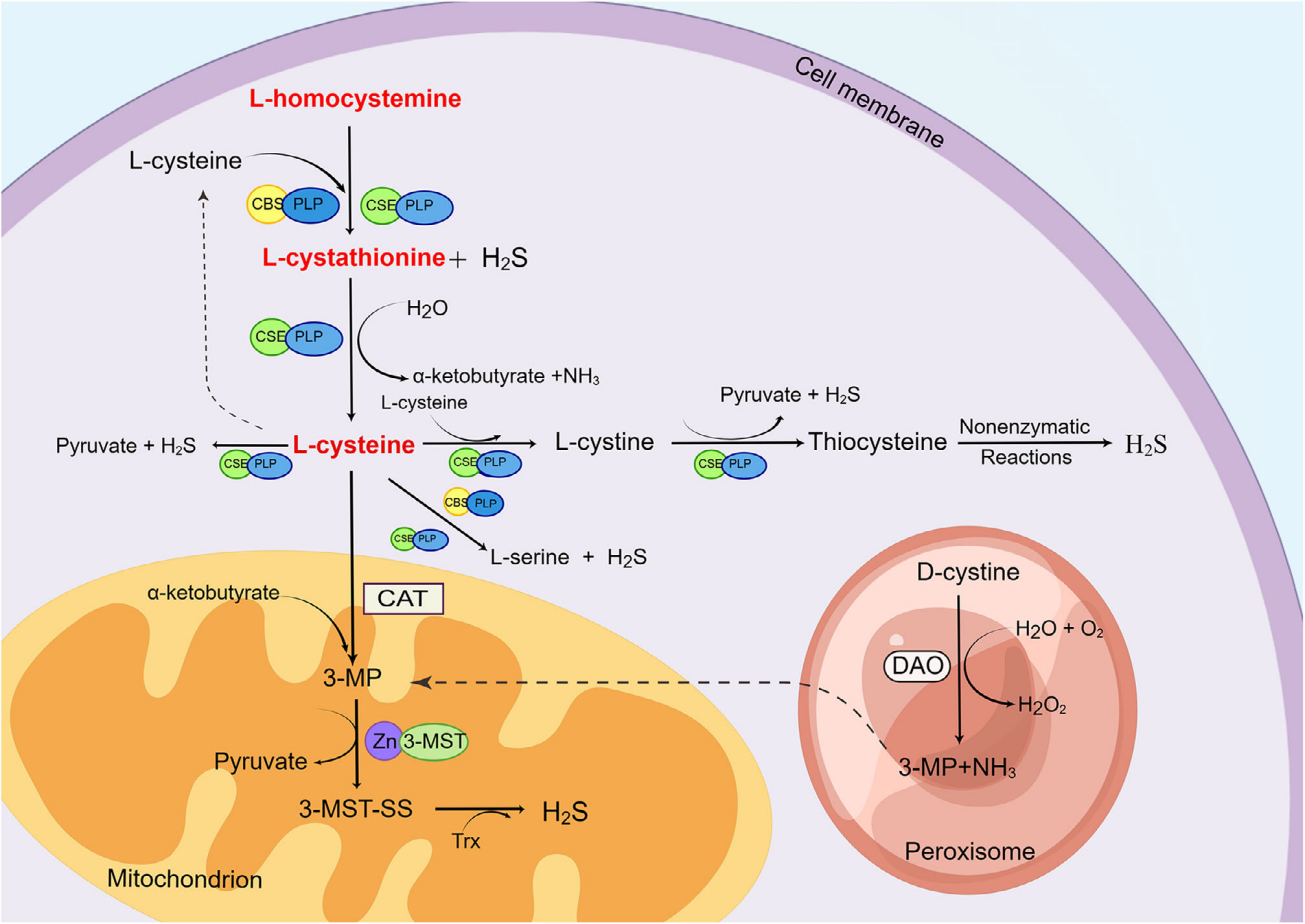
Enzyme catalysis of H_2_S production. Endogenous H_2_S can be produced by two ways: enzyme catalysis and nonenzyme catalysis. Enzyme catalysis is the main way and is catalyzed by four enzymes, such as CBS, CSE, 3‐MST and DAO. By Figdraw. H_2_S, hydrogen sulfide; CBS, cystathionine β‐synthase; CSE, cystathionine γ‐lyase; PLP, pyridoxal‐5′‐phosphate; 3‐MST, 3‐mercaptopyruvate sulfurtransferase; 3‐MP, 3‐methylpyridine; CAT, cysteine aminotransferase; DAO, D‐amino acid oxidase.

Both CBS and CSE use pyridoxal phosphate (also known as vitamin B6) as cofactors, and their concentrations vary in different tissues.^27^, ^28^ CBS mainly exists in the central nervous system (CNS) (cerebellum, hippocampus) and liver tissue.^29^ CSE mainly regulates H_2_S in the cardiovascular system and respiratory system.^15^ Only present in the cytoplasm, these two enzymes catalyze the conversion of homocysteine to cysteine, generating H_2_S through the reverse sulfur conversion pathway. Research has found that 3‐MST is an enzyme involved in endogenous H_2_S production.^30^ Unlike the two enzymes mentioned earlier, the cofactor of 3‐MST is zinc.^20^ It often cocatalyzes with cysteine aminotransferase (CAT) in mitochondria to produce H_2_S, L‐cysteine, and α‐Ketoglutaric acid generates 3‐mercaptopyruvate (3‐MP) under the catalysis of CAT, and then generates H_2_S and pyruvic acid under the action of 3‐MST.^31^ In 2013, Japanese scientists proposed a new enzyme catalysis pathway.^32^ This pathway occurs in the peroxisome. d‐Amino acid oxidase catalyzes d‐cysteine to produce 3‐MP, and then the product is transported to mitochondria through vesicles to participate in the next reaction.^33^, ^34^, ^35^ Endogenous H_2_S enzymatic generation pathway (as shown in Figure 1). Some studies have found that when the human body is under oxidative stress or hyperglycemia, the H_2_S produced through nonenzyme catalysis will significantly increase. In red blood cells, the reduction equivalent produced by glucose oxidation can be utilized to reduce elemental sulfur or polysulfides to H_2_S.^20^

### 2.3 Metabolism of endogenous H_2_S

**FIGURE 2:**
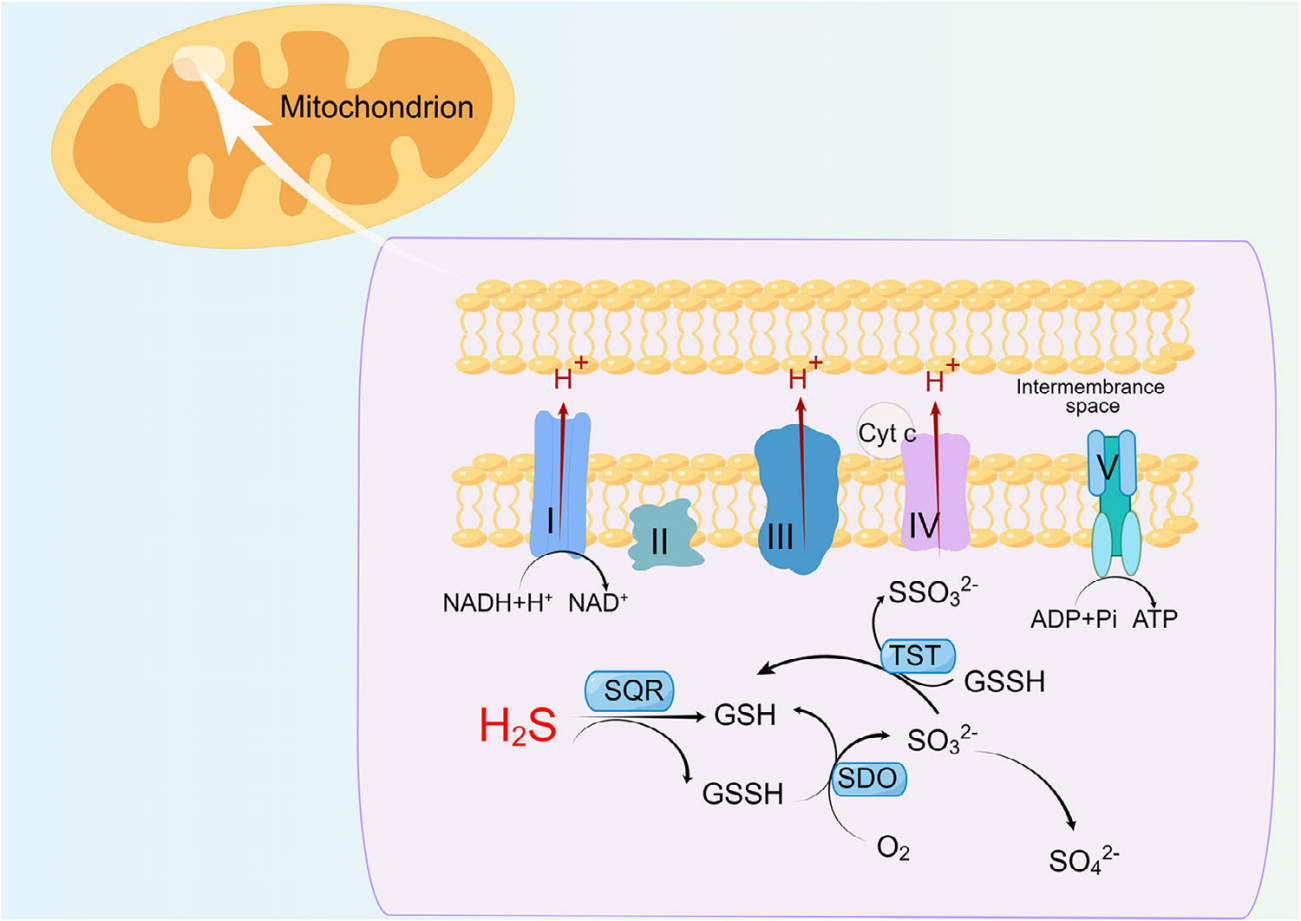
The oxidation pathway of endogenous H_2_S. The H_2_S oxidation pathway in mitochondria is mainly catalyzed by sulfuroquinone oxidoreductase. Finally, H_2_S is discharged from the body in the form of Thiosulfate or sulfate through this pathway. By Figdraw. ADP, adenosine diphosphate; ATP, adenosine triphosphate; Cyt *c*, cytochrome *c*; GSH, glutathione; GSSH, glutathione disulfide; H_2_S, hydrogen sulfide; NADH, nicotinamide adenine dinucleotide; SQR, sulfur quinone oxidoreductase; SDO, sulfide dioxygenase; TST, rhodanese.

In mammals, the excretion of H_2_S varies in different systems. In the respiratory tract, H_2_S is directly excreted as gaseous molecules. Through the urinary tract, H_2_S is mainly excreted as thiosulfate or free sulfate in the urine. However, in the gastrointestinal tract, most H_2_S is still excreted as free sulfide in the feces.^20^ H_2_S mainly has the following three metabolic pathways: (1) The elimination of H_2_S through the mitochondrial sulfide oxidation pathway, with sulfide quinone oxidoreductase (SQOR) being the key enzyme in this reaction (as shown in Figure 2). First, H_2_S is oxidized to thiosulfate under the catalysis of SQOR.^36^, ^37^ In this reaction, the primary sulfur acceptor is glutathione (GSH), and the resulting product is glutathione persulfide (GSSH).^38^, ^39^ In the next step of the reaction, rhodanese (or thiosulfate sulfurtransferase) plays a crucial role as a sulfur transferase that can further oxidize thiosulfate to sulfite or sulfate.^40^, ^41^ However, due to the rapid oxidation of sulfite to sulfate, H_2_S is ultimately expelled from the body in the form of thiosulfate or sulfate through this pathway.^20^, ^41^ (2) Research has found that methylation occurring in the cytoplasm is another metabolic pathway for H_2_S.^26^ Thiol‐S‐methyltransferase (TSMT) can catalyze the conversion of H_2_S to methyl mercaptan and dimethyl sulfide. TSMT is commonly present in cells in the human body but has high activity in mucosal cells of the colon and cecum.^42^ Compared with the sulfide oxidation pathway, the metabolic process of sulfide methylation is slower. In a study, the rate of sulfide methylation in mammalian colon mucosal cells was approximately 10,000 times slower than the oxidation rate of H_2_S.^43^ (3) H_2_S can be cleared by methemoglobin, metal‐containing, or sulfur‐containing macromolecules. Methemoglobin and myoglobin can promote the binding of H_2_S and iron by regulating the reactivity of iron, accelerating the oxidation rate of H_2_S.^44^

## 3 PHYSIOLOGICAL FUNCTIONS OF H_2_S

### 3.1 H_2_S and cardiovascular regulation

Initially, H_2_S was thought to have a similar role to NO in relaxing blood vessels. As it has been studied more intensively, H_2_S can maintain endothelial cell function by several mechanisms, such as increasing NO bioavailability and stabilizing the translation of endothelial nitric oxide synthase (eNOS).^45^ ^,^ ^46^ Early researchers found that H_2_S can cause concentration‐dependent vasodilation by opening adenosine triphosphate (ATP)‐sensitive potassium (K_ATP_) channels in vascular smooth muscle and decreasing extracellular calcium (Ca^2+^) in flow.^47^ Later experimental results have shown that glibenclamide can partially inhibit this vasodilation. Nevertheless, the molecular mechanism of how endogenous H_2_S activates K_ATP_ channels remains unclear, and recent studies may fill this gap. It was found that the K_ATP_ protein Kir6.1 undergoes S‐Sul hydration when CSE is overexpressed, and another study found that Kir6.1, which undergoes S‐sulfation at the Cys43 site, reduces the synthesis of ATP and enhances the function of the K_ATP_ channel, promoting vasodilation.^48^, ^49^ Meanwhile, H_2_S has been investigated as a possible endothelium‐derived relaxing factor, which combines with eNOS to produce S and NO that can activate transient receptor potential (TRP) ankyrin‐ 1 channels, leading to hyperpolarization and vasodilation in endothelial cells and smooth muscle cells.^49^ Therefore, H_2_S has potential therapeutic possibilities for targeting diseases of the vascular system, but more in‐depth mechanistic studies are still needed to determine this. In addition, various mechanisms have been implicated in H_2_S‐induced vasodilation, such as the protein kinase G (PKG) pathway, Cl^−^/HCO_3_^−^ channel, and TRP channel.^50^, ^51^, ^52^

H_2_S also plays a vital role in angiogenesis. Angiogenesis is an essential physiological process in mammals that maintains normal life activities, and it consists of several successive stages, among which the migration of vascular endothelial cells is most important. In earlier studies, researchers found that CSE inhibitors reduced the length of blood vessels in chicken chorioallantoic membrane.^53^ In another study, exogenous administration of sodium hydrosulfide (NaHS) promoted endothelial cell proliferation and migration, which may be related to the phosphoinositide 3‐kinase (PI3K)/protein kinase B (Akt) signaling pathway.^54^ However, these results were not strong evidence that endogenous H_2_S regulates angiogenesis. The researchers then did several more studies, and they found that mice knocked down for CSE had more rapid angiogenesis and wound healing in response to vascular endothelial growth factor (VEGF) stimulation compared with wild‐type mice.^53^ In terms of mechanisms, a growing number of studies have found that both H_2_S and NO play important roles in angiogenesis.^55^ For example, H_2_S and NO share a common downstream molecule, silent information regulator‐1 (SIRT1), the activation of which increases the expression levels of VEGF and cyclic guanosine 3′, 5′‐monophosphate (cGMP) and thus participates in the regulation of angiogenesis. In the available reports, the mechanisms by which H_2_S and NO promote cGMP expression are also different, with H_2_S degrading cGMP by inhibiting phosphodiesterase 5. However, NO promotes soluble guanylyl cyclase production of the cGMP.^56^ As described above, H_2_S activates Akt phosphorylation and may increase eNOS phosphorylation at the activation site Ser1177.^57^ Therefore, H_2_S is a physiologically important factor in angiogenesis.

### 3.2 H_2_S and neuromodulation

In an early study in 1989, researchers serendipitously discovered that H_2_S could be detected in the brains of rats even when NaHS was not administered and was prevalent in several regions of the brainstem, cerebellum, hippocampus, and striatum.^5^ Since then, researchers have studied the interactions between endogenous H_2_S and the nervous system. In 1996, researchers discovered an interesting phenomenon that endogenous H_2_S can enhance N‐methyl‐d‐aspartate (NMDA) receptors, thus contributing to learning and memory processes.^6^ They have shown that knockdown of CBS or 3‐MST leads to significant impairment of physiological memory formation in mice.^58^ Another study showed that direct infusion of various CBS inhibitors into the lateral amygdala impaired long‐term enhancement of this region, which led to inhibition of cued fear memory formation in rats.^59^ The concept that H_2_S participates in synaptic neurotransmission has existed since the early 21st century.^60^, ^61^ Investigators have demonstrated the involvement of CBS‐derived H_2_S in nucleus tractus solitarius excitability by using a combination of molecular and pharmacological approaches, providing evidence for the role of endogenous H_2_S in excitatory neurotransmission.^62^ This may indicate that H_2_S acts presynaptically and involves enhancing intracellular Ca^2+^ channels. Overall, most of the studies discussed above were performed in vitro using high (millimolar) NaHS concentrations and could not draw definitive conclusions about the potential physiological relevance of these effects. Therefore, future studies that aim to determine the physiological effects of H_2_S should be conducted using donors in the low micromolar range.

### 3.3 H_2_S and gastrointestinal regulation

In the gastrointestinal tract, under physiological conditions, H_2_S is produced endogenously and exogenously; endogenous is the enzyme origin we mentioned earlier since the two enzymes, CBS and CSE, are present in almost the entire gastrointestinal tract of mammals.^63^ Regarding the exogenous production of H_2_S, it is mainly the microbiota in the gastrointestinal tract responsible, with sulfate‐reducing bacteria being one of the leading producers. Microbial metabolism breaks down proteins in the gastrointestinal tract into amino acids, which include cysteine, H_2_S, and other sulfur‐containing compounds.^64^, ^65^ So, does H_2_S present in the gastrointestinal tract influence gastrointestinal motility? Past studies have given us the answer that the effect of H_2_S on gastrointestinal motility is nonunique. Generally, in vitro studies show that NaHS inhibits smooth muscle contraction in the gastrointestinal tract of different species. For example, in ex vivo experiments in rabbits, guinea pigs, mice, and rats, NaHS concentration‐dependently inhibited spontaneous and agonist‐induced contractions of the small bowel and colon, and ileal contractions in guinea pigs were slowly augmented using the inhibitor pregnancy‐associated glycoprotein (PAG).^63^, ^66^, ^67^, ^68^, ^69^, ^70^ However, NaHS has a dual effect on spontaneous contractions of gastric smooth muscle in guinea pigs and mice, that is, high concentrations of NaHS inhibit the amplitude of gastric smooth muscle contractions. In contrast, low concentrations increase basal tone.^71^, ^72^ From the above studies, we can find that the effects of H_2_S on smooth muscle may vary depending on the species and the location of the digestive tract, so the mechanisms also diverge. The inhibitory effects of H_2_S reported so far may be related to K_ATP_ channels, L‐type voltage‐dependent calcium channels, cGMP/PKG pathway, and sodium channels.^67^, ^73^, ^74^, ^75^, ^76^

### 3.4 H_2_S and inflammation

Among the many controversial areas of H_2_S research, the role of H_2_S in inflammatory processes is undoubtedly an example. H_2_S has been reported to have proinflammatory and anti‐inflammatory effects.^77^, ^78^, ^79^, ^80^, ^81^, ^82^ For example, the upregulation of CSE induced by lipopolysaccharide (LPS) or proinflammatory cytokines and the consequent increase in H_2_S production can be viewed as a proinflammatory effect or an anti‐inflammatory response as a compensatory protective mechanism.^78^, ^83^ One study found that injection of the H_2_S donor NaHS in rats inhibited leukocyte infiltration and adhesion.^84^ On the other hand, H_2_S synthesis inhibitors increase leukocyte adhesion, leukocyte infiltration, and edema formation. In animals suffering from acute lung injury caused by burns and smoke inhalation tissue, tissue interleukin (IL)‐1 levels were decreased, IL‐10 levels were increased in inflamed lungs, and protein oxidation was attenuated after NaHS injection.^82^ Experimental evidence suggests that H_2_S is a proinflammatory factor in various animal models, including acute pancreatitis, LPS‐induced endotoxemia, cecum ligation, and puncture‐induced sepsis.^78^, ^85^ One possible scenario for the proinflammatory mechanism of H_2_S is that H_2_S stimulates sensory nerve endings, releasing endogenous tachykinins such as substance P, calcitonin gene‐related peptide, and neurokinin A, leading to neurogenic inflammation. Many factors are involved in determining whether H_2_S is proinflammatory or anti‐inflammatory, and the concentration of H_2_S and route of administration may produce different inflammatory outcomes.

### 3.5 H_2_S and aging

The earliest suggestion that the endogenous H_2_S pathway may be regulated during aging was made by researchers Finkelstein and Benevenga, who found that the metabolic capacity of hepatic homogenates to produce volatile sulfur compounds (H_2_S and/or methanethiols) from 3‐methylthiopropionate was a product of the metabolism transamination pathway of methionine. All three enzymes, CBS, CSE, and 3‐MST, have been reported to show significant age‐related downregulation in the brains of aging rats.^86^ Although some H_2_S‐producing enzymes may be upregulated in some tissues of aging animal models, plasma H_2_S levels decline with age in rats and mice; moreover, H_2_S donation produces organ‐protective and beneficial effects.^87^, ^88^, ^89^, ^90^ A large body of experimental evidence suggests that endogenous or exogenous H_2_S can modulate many core mechanisms implicated in the aging process. These mechanisms include regulation of genome stability, telomere maintenance, epigenetic regulatory mechanisms, mitochondrial function/dysfunction, stem cell depletion, and protein inhibitory processes.

## 4 PATHOPHYSIOLOGIC ROLE OF H_2_S IN DISEASE

### 4.1 H_2_S and IRI

According to the progression of diseases, IRI can be divided into two stages: ischemia and reperfusion. It is generally believed that the degree of cell dysfunction, injury, and necrosis is related to the severity and duration of ischemia. Therefore, the main idea for treating IRI is to restore blood flow to the ischemic site as soon as possible.^91^ However, the sensitivity of different organs to ischemic manifestations also varies, such as the brain, heart, and other organs with poor tolerance to ischemia and hypoxia, and differences in organ tolerance can also affect the degree of cell damage. In addition, although the reperfusion recovery can provide oxygen and nutrients to cells, it will further strengthen the damage after ischemia, activate cell death and immune response, and so on.^92^ On the other hand, inflammatory mediators will also be transported to the distal organs with the recovery of reperfusion, which is also the reason for multiorgan failure in the later stage of IRI.^93^, ^94^, ^95^ IRI is a dynamic process with significant organ differences, so a deeper understanding of its molecular mechanisms can help us find better treatment methods.

#### 4.1.1 Ca^2+^ overload

**FIGURE 3:**
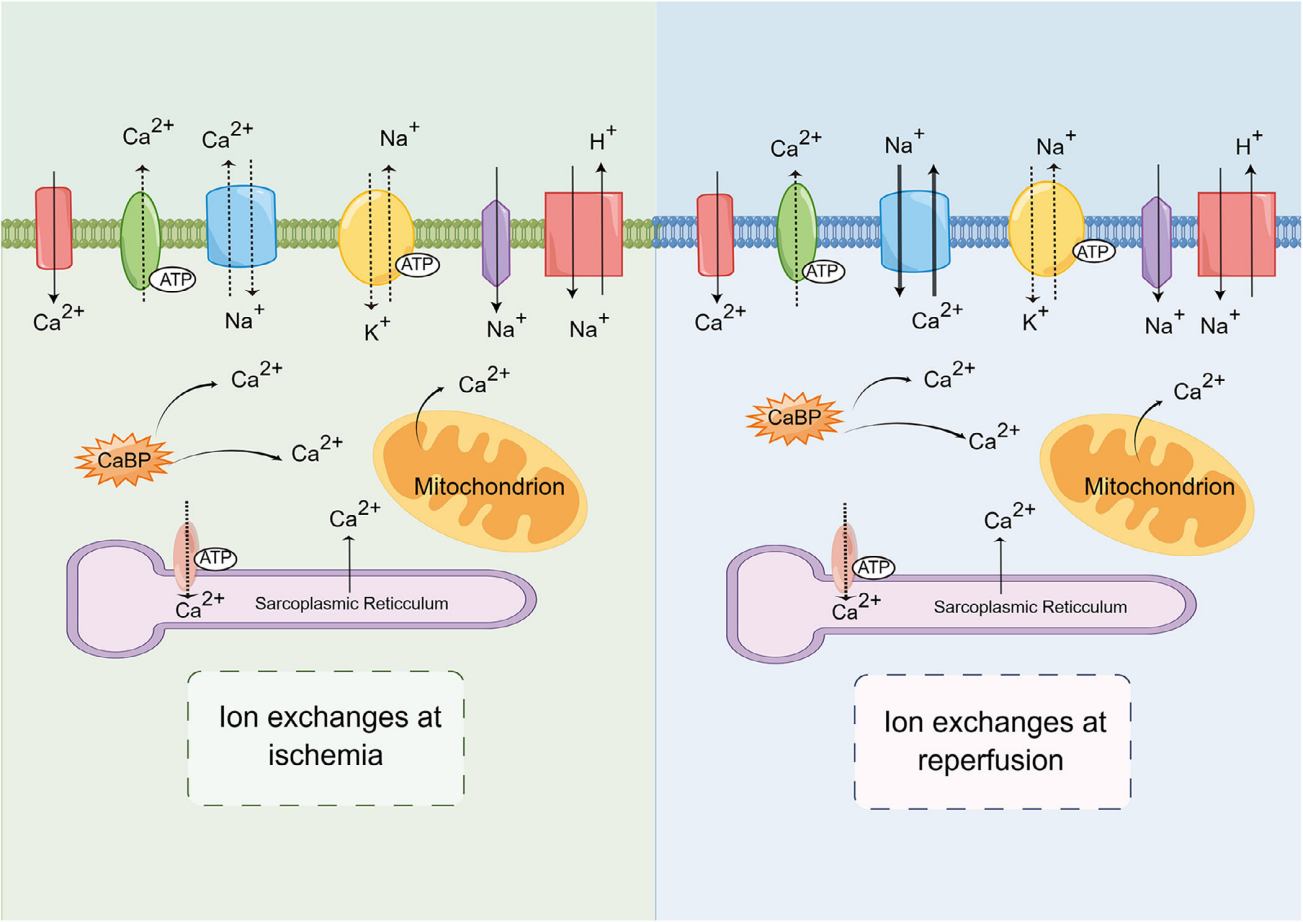
The mechanism of ion exchange leading to Ca^2+^ overload during IRI. In IRI, abnormalities in Na^+^–Ca^2+^ exchange are associated with the following three aspects. First, high intracellular Na^+^ directly activates natriuretic proteins during ischemic injury. Second, high intracellular H^+^ decreases pH, which indirectly activates natriuretic proteins. At last, activation of PKC and increased release of catecholamines during reperfusion further promotes Na^+^–Ca^2+^ exchange. By Figdraw. CaBP, calcium binding protein; Ca^2+^, calcium ion; H^+^, hydrogen ion; Na^+^, sodium ion; K^+^, potassium ion.

When ischemia occurs, ATP in cells is rapidly depleted, ATP synthesis decreases, sodium pump activity decreases, intracellular Na^+^ content increases, and sodium–Ca^2+^ exchange proteins are activated, leading to reverse transport of Na^+^ to the extracellular space and an increase in intracellular Ca^2+^.^96^, ^97^ On the other hand, due to hypoxia and anaerobic metabolism, the production of H^+^ increases, and the pH of extracellular fluid and cytoplasm decreases. When tissue perfusion resumes, the pH of extracellular fluid increases, but the pH of cytoplasm is still very low. In order to reduce the accumulation of H^+^ in cells, H^+^–Na^+^ exchange protein and Na^+^–Ca^2+^ exchange protein is activated, increasing Ca^2+^ overload.^96^ When the body is in a state of stress, releasing a large amount of catecholamines activates protein kinase C (PKC) through a signaling pathway, promotes H^+^–Na^+^ exchange, and also increases intracellular Ca^2+^. Due to the massive accumulation of Ca^2+^, the endoplasmic reticulum and mitochondria damage intensifies. The complete opening of the mitochondrial mitochondrial permeability transition pore (mPTP) will have a more negative impact on cells.^98^ Figure 3 illustrates the mechanism of Ca^2+^ overload during the ischemia and reperfusion phases caused by ion exchange.

#### 4.1.2 ROS

When ischemic tissue undergoes reperfusion, blood brings oxygen and nutrients to the tissue. At the same time, due to the low concentration of antioxidants in cells, the production of reactive oxygen species (ROS) increases. In the I/R process of biology, ROS will be produced in many ways, including mitochondrial electron transfer chain (ETC), xanthine oxidase system (XOD), NADPH oxidase system, nitric oxide synthase (NOS), and so on.^99^ The first three are related to oxidative stress in multiple organs, such as the heart, brain, lungs, liver, pancreas, kidneys, and gastrointestinal tract.^100^ NOS mainly acts as an oxidative stress factor in vascular endothelial cells.^101^ During the metabolism of normal mitochondria, the respiratory chain complex on the inner mitochondrial membrane can produce a small amount of ROS.^102^ As mentioned earlier, when IRI occurs due to hypoxia, changes in ATP, pH, and Ca^2+^ overload occur in cells, which can lead to mitochondrial damage and produce more ROS. However, ROS further exacerbates oxidative stress, leading to a vicious cycle of cells.^96^, ^102^

The XOD system is an important pathway for ROS production. Under ischemia, ATP synthesis is reduced, and xanthine dehydrogenase is converted into XOD. At the same time, ATP degradation products (ADP, AMP, hypoxanthine) accumulate. When reperfusion is resumed, a large amount of oxygen molecules enters the tissue with the blood. XOD catalyzes the conversion of hypoxanthine into xanthine and uric acid, producing a large amount of ROS.^103^ The oxygen free radicals generated by this pathway have chemotactic effects, attracting and activating many white blood cells to aggregate. When the tissue resumes its oxygen supply, the activated white blood cells’ oxygen consumption increases sharply, producing a large amount of oxygen free radicals, causing cell damage.

The NOx/Deox family of NADPH oxidase mainly includes seven subtypes, such as Nox‐1, Nox‐2, Nox‐3, Nox‐4, Nox‐5, Duox‐1, and Duox‐2; these enzymes have the ability to produce ROS.^104^ Under hypoxic conditions, hypoxia inhibitory factor‐1α. Promote the activation of NOX enzyme, and after reperfusion, cells will release some chemical factors to activate further NADPH oxidases, such as phospholipase A2, tumor necrosis factor‐α (TNF‐α), interleukin‐1 beta (IL‐1β), interferon‐γ, angiotensin II (Ang II), and so on. Overexpression of NADPH oxidase after activation enhances ROS production.^105^ ^106^

In addition to the pathways above, NOS is also an important pathway for generating ROS. Tetrahydrobiopterin (BH4) is a cofactor of the NOS enzyme. In IRI, oxidative stress oxidizes BH4 to BH2, decreasing the BH4 cell level and uncoupling NOS, thereby promoting ROS production.^107^

#### 4.1.3 Cell death

The IRI process of organisms is dynamic, and the mechanism for producing ROS is also complex. The ROS produced by the above pathways may accumulate in cells during the ischemic stage and inhibit antioxidants. However, after tissue restoration of blood supply, if ischemia is severe, ROS‐induced oxidative stress may further cause cell damage and even cell death.^108^

Apoptosis is a process of programmed cell death, mainly caused by Ca^2+^ overload and ROS activation in IRI. Two cell apoptosis pathways can interact with the endogenous and exogenous apoptosis pathways. The endogenous pathway is also known as the mitochondrial pathway. In the cells injured by ischemia/reperfusion, a large amount of calcium influx and ROS production will cause the opening of mitochondrial mPTP and the activation of apoptosis promoting B‐cell lymphoma‐2 (Bcl‐2) family, increase the permeability of mitochondrial membrane, release cytochrome *c* into the cytoplasm, and then combine with apoptosis protease activating factor 1 (APAF‐1) to activate Caspase‐9 and form a complex, and then trigger the apoptosis cascade reaction to promote apoptosis.^109^ The exogenous pathway, or the death receptor pathway, is mainly activated by death factors or receptors. Important death factors include TNF‐α, Fas ligands, tumor necrosis factor (TNF)‐related apoptosis‐inducing ligand, and tumor necrosis factor‐like cytokine 1A. As mentioned earlier, during IRI, reperfusion cells release TNF‐α and activate the c‐Jun N‐terminal kinase (JNK) pathway to stimulate the production of ROS. The accompanying oxidative stress will further stabilize the phosphorylation of JNK and accelerate cell death.^110^ If TNF‐α persistent increase will induce the TNFR‐related death domain (TRADD) to combine with it to synthesize TNF α‐TRADD. TNF α‐TRADD and Fas FADD can induce and activate caspase 8 and 10, then enzymolysis activates downstream caspase 3, 6, and 10, and then starts the cell apoptosis.^111^ However, cell apoptosis in IRI is not as typical as the necrosis mentioned below.

Necrotizing apoptosis is also programmed cell death, but its impact on organisms completely differs from previous cell apoptosis. The main characteristics of necrotic apoptosis are cell swelling, organelle disintegration, and leakage of intracellular components, which often cause severe inflammatory reactions in ischemic tissues.^112^, ^113^ As a regulatory mode of death, the main factors triggering necrotic apoptosis are the interacting serine threonine‐kinase 3 (RIPK3) and mixed lineage kinase‐like domain (MLKL).^114^ RIP3 can enable TNF‐α driven cell death changes from apoptosis to necrotic apoptosis. When Caspase‐8 is depleted or the inhibitor of apoptosis protein is deficient, TNF receptor I will promote necrotic apoptosis.^113^ The assembly of the necrotic complex is mainly related to RIPK1/RIPK3 interaction and MLKL activation. RIPK3 induces phosphorylation of MLKL, leading to oligomerization and translocation of MLKL to the lobules within the plasma membrane, ultimately increasing plasma membrane permeability and cell death.^114^ ROS‐induced DNA damage also promotes the formation of necrotic complexes by activating poly (ADP‐ribose) polymerase (PARP), further accelerating cell death. Due to the close relationship between necrotic apoptosis and the occurrence of inflammation in the human body, understanding the molecular mechanism and pathophysiological significance of necrotic apoptosis remains an essential goal of therapeutic IRI research.

**FIGURE 4:**
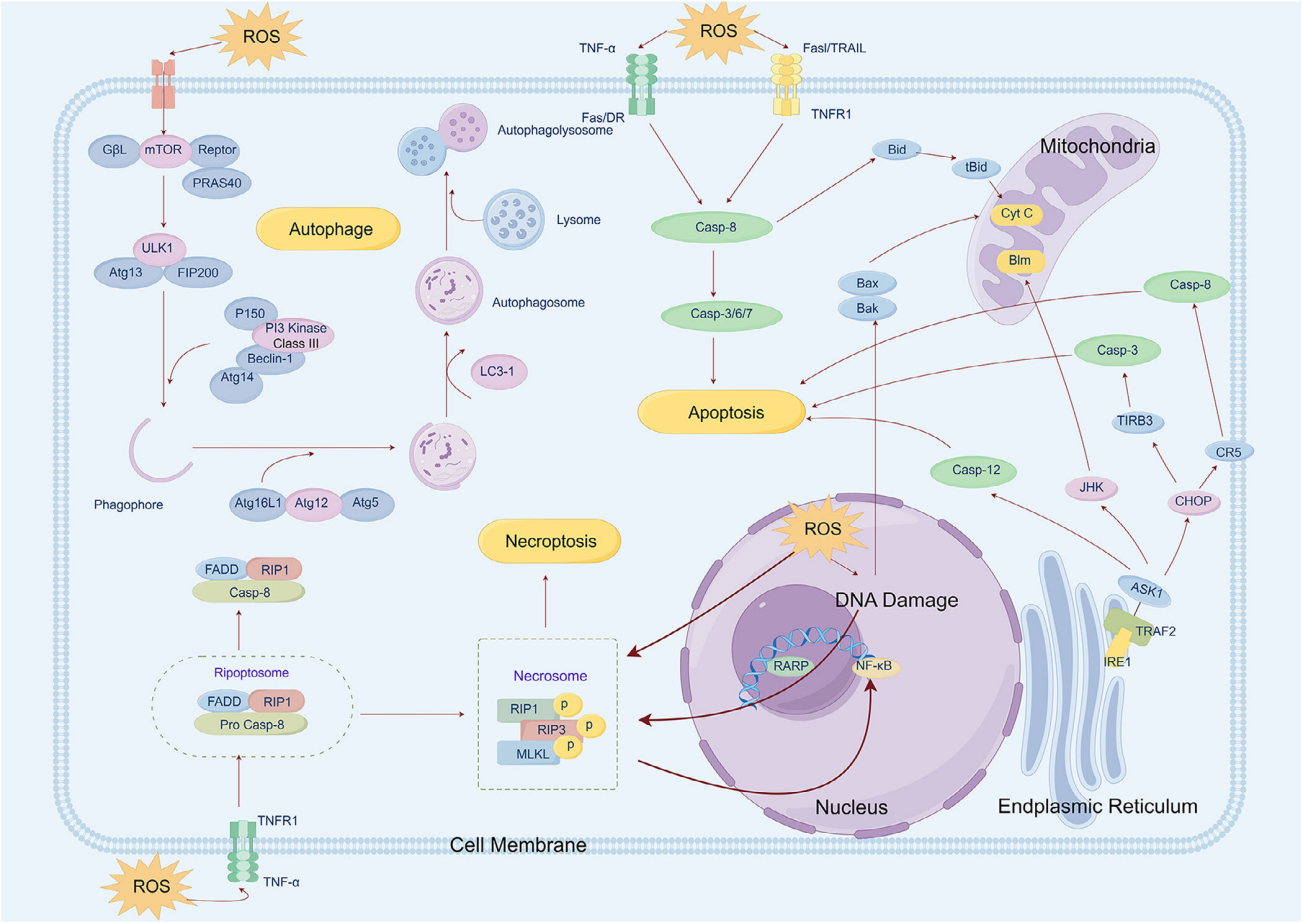
ROS‐induced cell death mechanism. ROS induced oxidative stress may further cause cell damage, even cell death, such as apoptosis, autophagy, and programmed cell death. By Figdraw. APAF‐1, apoptosis protease activating factor 1; Cyt *c*, cytochrome *c*; FADD, Fas‐associating protein with a novel death domain; MLKL, mixed lineage kinase like domain; NF‐κB, nuclear factor‐kappa B; RARP, poly(ADP‐ribose) polymerase; ROS, reactive oxygen species; TNF‐α, tumor necrosis factor‐α; TRADD, TNFR‐related death domain.

The role of autophagy in IRI is bidirectional, which can both protect cells and disrupt them. Appropriate mitochondrial autophagy can clear partially damaged mitochondria during ischemia and reduce subsequent damage.^115^ During the reperfusion phase, the levels of Ca^2+^ and ROS increase in the cells, while oxidative stress inhibits the activity of rapamycin mechanistic target of rapamycin (mTOR), leading to the formation of UNC51‐like kinase 1 complexes and PI3K III class, which induce autophagy. However, autophagy cannot clear all damaged mitochondria, and when autophagy clearance capacity is exceeded, it can lead to cell death.^116^, ^117^ Figure 4 provides a detailed description of ROS‐induced cell death mechanism.

#### 4.1.4 Inflammation

During reperfusion, producing a large amount of ROS activates the nuclear factor‐κappa B (NF‐κB) gene, further stimulating the secretion of TNF‐α by endothelial cells and macrophages.^118^ Conversely, TNF‐α can induce cell apoptosis through a sphingosine‐dependent mechanism. On the other hand, it can also cause leukocyte infiltration in damaged tissues, increase the permeability of vascular endothelial cells, produce a no‐reflow phenomenon, and aggravate reperfusion injury.^119^, ^120^ At the same time, activated cells release many inflammatory factors, such as IL‐1, IL‐6, IL‐8, IL‐10, IL‐18, and so on.^121^

#### 4.1.5 Nephroprotective effects of H_2_S

Renal IRI is a major predisposing factor for the development and progression of acute kidney injury (AKI).^122^, ^123^ AKI is a complex clinical syndrome characterized by a rapid decline in renal function, such as decreased glomerular filtration rate (GFR) with increased creatinine and urea nitrogen, water‐electrolyte disturbances, acid–base imbalance, oliguria, or even anuria.^124^, ^125^AKI is often associated with severe complications, and the high mortality rate places a significant burden on the healthcare system.^126^ Some recent studies have shown that H_2_S can improve renal function during IRI to prevent AKI, associated with decreased ROS expression.^127^, ^128^, ^129^ In addition to antioxidant effects, H_2_S may exert renoprotective effects through several other mechanisms. First, H_2_S can induce vascular relaxation by opening K_ATP_ channels in endothelial and renal vascular smooth muscle cells, thereby increasing renal blood flow.^47^, ^130^ Second, H_2_S can potentially protect renal function by inhibiting Ang II in the renin‐angiotensin‐aldosterone system.^131^ In addition, some investigators have also found that AP39, a mitochondrial‐targeting H_2_S donor, can reduce ROS levels, protect mitochondrial function, and reduce renal epithelial cell injury. However, this protective effect is dose‐dependent.^81^, ^132^

#### 4.1.6 Hepatoprotective effects of H_2_S

Ischemic liver tissue is extremely susceptible to more severe liver dysfunction and failure after reperfusion occurs.^133^, ^134^ More seriously, hepatic ischemia–reperfusion (HIR) can also affect the success of liver resection or transplantation and increase the risk of death for the operator.^133^, ^135^ The risk of death is increased. There is a lot of experimental data to demonstrate that H_2_S can effectively protect liver tissue in HIRI and is expected to be a new way to reduce the morbidity and mortality of HIRI complications.^136^, ^137^, ^138^ Some experiments have shown that the expression levels of endogenous H_2_S and CSE are elevated in the tissues of HIR rats, and the researchers speculate that this may be due to the self‐protective response of the organism induced by HIR. Meanwhile, after using the exogenous H_2_S donor NaHS in HIR rats, the investigators found that NaHS could attenuate IRI‐induced liver injury^136^, ^139^ At present, there has been a large amount of data demonstrating that H_2_S can play a role in reducing liver injury through various mechanisms, such as inhibition of oxidative stress, antiapoptosis, anti‐inflammation, protection of mitochondrial function, and regulation of autophagy.^140^, ^141^, ^142^, ^143^, ^144^, ^145^ However, it has also been found that endogenous H_2_S may exacerbate HIR‐induced liver injury in the context of insulin resistance, so H_2_S should be used with caution in this situation.^146^

#### 4.1.7 Retinal protective effect of H_2_S

IRI to the retina is the cause of many retinal vascular diseases, such as diabetic retinopathy (DR), glaucoma, retinal artery occlusion, and so on.^147^ It is mainly caused by generating and accumulating large amounts of ROS during ischemia and reperfusion. It causes a series of oxidative stress and inflammatory responses that promote irreversible damage to retinal ganglion cells, which may eventually lead to vision loss or even blindness.^148^ In a study more than a decade ago, researchers injected an H_2_S donor (ACS67) into the vitreous humor of rats with retinal IRI. Subsequently, they found that ACS67 could regulate GSH levels and inhibit apoptosis of retinal ganglion cell‐5 (RGC‐5) cells induced by oxidative stress, thus exerting a protective effect.^149^ Another experiment found that direct inhalation of H_2_S for pretreatment prior to retinal IRI in rats reduced the mortality of RGC.^150^ In a 2016 study, it was first proposed that enzymes involved in the generation of H_2_S and related pathways are activated during retinal IRI and may have the ability to induce retinal neovascularization.^151^ In addition, H_2_S may also protect retinal ganglion cells by inhibiting the production of inflammatory factors and activating signaling pathways involved in mediating the protection of mitochondrial function and diastolic vascularity.^152^, ^153^, ^154^

#### 4.1.8 Testicular protective effect of H_2_S

Testicular torsion is a urological emergency that occurs in children and requires immediate surgical treatment; however, despite successful surgical intervention, the incidence of associated complications (such as testicular atrophy and infertility) ranges from 40 to 60%.^155^, ^156^ Postoperative IRI is the main cause of testicular damage, and previous studies have demonstrated that testicular IRI is closely related to excessive production of ROS, with subsequent massive production of inflammatory factors, oxidative stress, and apoptosis further exacerbating tissue damage.^157^, ^158^ The subsequent high production of inflammatory factors, oxidative stress, and apoptosis further aggravate the tissue damage. In the last 2 years, studies have revealed that H_2_S may have potential therapeutic effects in protecting testicular tissue.^159^, ^160^ Bozkurt et al.^160^ first investigated the role of H_2_S in IRI in testicular torsion. They found that H_2_S administration inhibited oxidative stress and suppressed the expression of TNF‐α, APAF‐1, and iNOS to reduce tissue damage.^160^ Myeloperoxidase (MPO), malonaldehyde, and advanced oxidation protein products (AOPP) are markers of lipid peroxidation, and Yuksel et al.^159^ found that NaHS could effectively reduce the expression levels of MPO and AOPP. Meanwhile, Johnson scores were significantly higher in the H_2_S administration group, suggesting that H_2_S can improve spermatogenic function in IRI testes.^159^ However, there are still relatively few related studies. The mechanism of the protective effect of H_2_S in testicular IRI is still unclear, and we need to conduct more in‐depth studies.

#### 4.1.9 H_2_S and organ transplantation

Organ transplantation is the treatment modality of choice for organ failure or severe lesions. The current clinically accepted standard for transplantation is static cold storage (SCS), which essentially involves soaking the donor organ in a variety of preservation solutions, such as University of Wisconsin (UW) solution or histidine–tryptophan–ketoglutaratesolution, and subsequently storing it on ice at 4°C.^161^ Short‐term SCS reduces the graft donor metabolic demand and cellular activity; however, long‐term SCS leads to severe cold IRI, which may cause severe rejection and reduce graft success.^162^, ^163^, ^164^

Researchers have tried adding additives to the graft preservation solution to discourage cold IRI. Among them, richer results have been achieved in adding H_2_S donors. Previous studies have shown that supplementing graft preservation fluids with appropriate doses of H_2_S donors such as NaHS, 10‐oxo‐10‐(4‐(3‐thioxo‐3H‐1,2‐dithiol‐5yl)phenoxy)decyl (AP39), and morpholin‐4‐ium 4 methoxyphenyl (morpholino) phosphinodithioate (GYY4137) can reduce the damage of IRI to kidney donors in rats and pigs and improve donor function.^128^, ^165^, ^166^, ^167^, ^168^ At the same time, H_2_S donors protect other donor organs from cold IRI, such as the heart, liver, lungs, and pancreas.^169^, ^170^, ^171^, ^172^ This protective function mainly relies on antioxidant, apoptosis, anti‐inflammatory, and vascular mechanisms. So far, in the study of H_2_S donor amelioration of cold IRI, AP39 has shown a high potential to target the mitochondria of donor organs and promote H_2_S entry. Nevertheless, AP39 is an experimental donor and is not clinically licensed, so it is important to find an H_2_S donor that is clinically licensed, which will further the process of moving H_2_S donors from the lab to the clinic. Previously, we mentioned sodium thiosulfate‐supplemented (STS) as an antidote that the United States Food and Drug Administration (US FDA) had approved. Some researchers have found that supplementation of STS in the UW solution in which kidneys are stored attenuates tubular necrosis and apoptosis, increases transplant survival, and improves transplant function.^173^ However, there are fewer reports on the inhibition of cold IRI by STS in SCS, and the specific molecular mechanisms still need to be determined. However, adding STS to the preservation solution is a more economical and convenient method, preventing cold IRI in SCS and improving the grafting success rate.

### 4.2 H_2_S and cardiovascular health

#### 4.2.1 Anti‐inflammatory effects of H_2_S in the cardiovascular system

Inflammation is another hallmark of endothelial dysfunction, and under pathological conditions, transcriptional activation of inflammatory adhesion factors leads to leukocyte and macrophage enrichment of the vascular lining, which induces a range of cardiovascular diseases.^174^ It has been reported in recent years that H_2_S may be an inflammatory modulator and play an anti‐inflammatory role in the cardiovascular system.^175^, ^176^ In a 2006 study, we reported that blocking endogenous H_2_S synthesis promoted leukocyte adhesion, which the administration of exogenous H_2_S donors conversely inhibited, and that this may be achieved by activating K_ATP_ channels.^84^ Later reports support the idea that H_2_S has an anti‐inflammatory effect. It has been reported that the endogenous H_2_S synthase CSE may be involved in the pathogenesis of atherosclerosis. When specifically absent, CSE enhances monocyte adhesion and accelerates endothelial damage and the atherosclerotic process. It is noteworthy that the use of H_2_S donors had the opposite effect. The underlying mechanism may be related to CSE‐H_2_S‐induced persulfuration at the Cys‐13 position.^177^ Another study in the same year demonstrated the anti‐inflammatory ability of H_2_S from a different perspective, as the investigators found that the H_2_S donors NaHS and GYY4137 increased the expression of mRNA for Sirtuin‐1 in arteries and induced its persulfuration, thereby inhibiting arterial inflammation and AS.^178^ TNF‐α has an obvious proinflammatory effect, and it can promote vascular inflammation by inducing endothelial cells to produce adhesion mediators such as monocyte chemotactic protein‐1 (MCP‐1). It has been found that H_2_S can inhibit the shedding of TNF‐α and the release of MCP‐1, thereby inhibiting inflammation.^179^ In addition to the above mechanisms, H_2_S has been found to exert anti‐inflammatory effects through various signaling pathways, such as inhibiting the JNK/NF‐κB signaling pathway and preventing the activation of NLPR3 inflammasome.^180^, ^181^, ^182^

#### 4.2.2 Myocardial protective effect of H_2_S

When the blood perfusion and oxygen supply of the myocardium are severely reduced, extensive cell death may occur, leading to myocardial infarction.^183^, ^184^ As mentioned earlier, although restoring blood supply can alleviate ischemia to a certain extent, it can also lead to a series of more serious reactions, such as oxidative stress, cell damage, and even death. In current research on IRI, increasing evidence suggests that endogenous H_2_S regulation or exogenous H_2_S donors may be involved in the pathogenesis of ischemic cardiomyopathy, improving cardiac function, controlling structural lesions, and reducing related complications.^185^, ^186^, ^187^ We found that H_2_S may have a protective effect on myocardial cells through the following five mechanisms.

Massively production of ROS during reperfusion is the main cause of oxidative stress responses. However, H_2_S can inhibit its production by regulating ROS signaling pathways, such as inhibiting NF‐κB and JAK2–STAT3 pathways to reduce ROS levels.^188^ At the same time, H_2_S can also increase the expression levels of superoxide dismutase (SOD) and GSH in IRI tissues, both of which are antioxidant enzymes that protect cardiomyocytes by converting peroxides (H_2_O_2_).^16^, ^189^ In addition, H_2_S can promote nuclear translocation of nuclear‐factor‐E2‐related factor‐2 (Nrf2), an important antioxidant transcription factor that increases transcription of antioxidant proteins and reduces apoptosis and mitochondrial damage.^190^

In the process of apoptosis, Bcl‐2 plays a crucial role. It has been reported that H_2_S is able to reduce the proportion of apoptotic cells in the myocardium of IRI mice by upregulating the expression level of Bcl‐2 and decreasing the expression of Bax and cysteine 3.^191^ Apoptotic proteins (IAPs) can block the apoptotic cascade response, and it has been found that H_2_S can inhibit apoptosis by affecting the phosphorylation of IAPs and cysteine aspartase recruitment structural domains.

H_2_S protects cardiomyocytes through a bidirectional action in autophagy.^192^ Tissue or cellular ischemia leads to the development of cellular autophagy, and moderate cellular autophagy facilitates the repair of damaged cells. It has been demonstrated that H_2_S can exert cytoprotective effects by promoting autophagy, which is associated with NLRP3 inflammatory vesicles. Excessive autophagy exacerbates cellular IRI, and H_2_S can activate the PI3K/serum glucocorticoid‐regulated kinase 1 (SGK1)/glycogen kinase synthase 3β (GSK3β) signaling pathway and PI3K/Akt/mTOR signaling pathway to inhibit autophagy and provide protection for IRI cardiomyocytes.^193^, ^194^

In the current study, H_2_S can inhibit the inflammatory response of cardiomyocytes, which is one of the important mechanisms for its cardioprotective effect.^195^, ^196^ In earlier years, some researchers found that certain H_2_S donors can reduce leukocyte adhesion and infiltration, and this effect seems to be related to the activation of K_ATP_ channels.^84^, ^197^ In addition, administration of H_2_S treatment before the ischemic tissue regains blood supply also prevents the activation of NF‐κB and reduces the production of proinflammatory mediators, with the most significant reduction of IL‐1 and IL‐6.^198^, ^199^ Increased expression levels of TNF‐α during reperfusion promote interaction between leukocytes and endothelial cells, resulting in increased infiltration of inflammatory cells in the IRI region of the myocardium, which leads to more severe myocardial injury, so inhibition of TNF‐α expression may attenuate myocardial injury. It has been found that H_2_S can inhibit the adhesion of inflammatory cells and release associated inflammatory factors caused by TNF‐α activation, significantly reducing the expression levels of MCP‐1, adhesion factors, and so on.^179^

The role of mitochondria is particularly important in mammalian growth and development, providing energy for the basic metabolism of the body. When myocardial IRI occurs, the function of mitochondria is severely impaired, leading to further myocardial damage. It has been found that NaHS can reduce mitochondrial malondialdehyde levels in ischemic cardiomyocytes while elevating the activities of SOD and glutathione peroxidase, allowing the preservation of mitochondrial function.^200^ In addition, H_2_S also increased the efficiency of complexes I and II of the oxidative respiratory chain in mitochondria. It inhibited cytochrome oxidase, reducing the metabolism of cardiomyocytes to a preconditioned state, thereby reducing cardiomyocyte damage.^201^, ^202^

### 4.3 H_2_S and the nervous system

#### 4.3.1 H_2_S and neurodegenerative diseases

H_2_S plays an important role in the CNS. In early times, H_2_S was recognized as a neuromodulator that played a role in enhancing long‐term memory in the hippocampal region of the brain in particular.^6^ Several studies have found that abnormal expression levels of endogenous H_2_S and the transsulfuration pathway may be associated with neurodegenerative diseases. ND, which includes Alzheimer's disease (AD), Parkinson's disease, Huntington's chorea, amyotrophic lateral sclerosis, and spinal cerebellar ataxia, is characterized by a pathological condition in which proteins are misfolded and aggregated, resulting in neuronal damage or even loss, which can in turn lead to cognitive and motor dysfunction.^203^, ^204^ We mentioned above that CSE and 3‐MST are predominantly found in neurons, and several studies have reported reduced or absent CSE expression in different NDs, with or without reduced protein persulfate translation.^86^, ^205^, ^206^ Oversulfuration modification may be a potential redox switch, and a growing number of studies suggest that H_2_S‐mediated oversulfuration modification may be involved in regulating oxidative stress, apoptosis, autophagy, and other processes to maintain normal physiological functions of cells.^16^, ^207^

The current study found that appropriate concentrations of H_2_S exert neuroprotective effects in ND, and in AD, phosphorylated Tau protein aggregates and misfolded β‐amyloid are its main features.^208^ H_2_S has been shown to cause peroxidative modification of GSK3β and inhibit phosphorylation of Tau proteins. At the same time, H_2_S has been shown to inhibit the expression of amyloid precursor protein cleaving enzyme 1 (BACE1), which, in turn, reduces β‐amyloid activity and prevents the development of AD.^206^, ^209^ In Parkinson's disease, H_2_S has been found to sulfurize the Parkin protein to promote E3 ligase expression and also induces peroxisulfuric modification of SIRT1 to increase mitochondrial activity, reduce neuronal damage, and further exert neuroprotective effects on neuronal cells.^205^, ^210^ H_2_S‐induced S‐sulfonylation of Nrf2 in Huntington's disease inhibits oxidative stress, thereby promoting autophagy of misfolded proteins and suppressing Huntington's disease progression.^211^ In summary, H_2_S has the role of a neuromodulatory transmitter. It can exert anti‐inflammatory, antiapoptotic, and antioxidative stress effects in neuronal cells, so it can be used as a potential neuroprotective agent in neurodegenerative diseases. However, high concentrations of H_2_S accelerate the progression of ND and aggravate neuronal cell damage. Therefore, our attitude regarding the research on using H_2_S as a neuroprotective agent should be cautious and in‐depth.

#### 4.3.2 Neuroprotective effects of H_2_S

**FIGURE 5:**
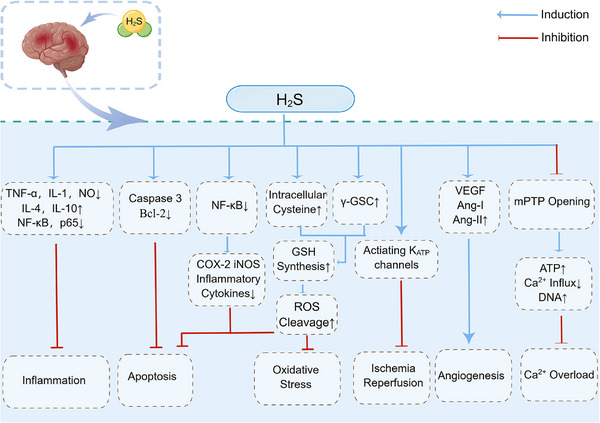
Mechanisms of neuroprotection exerted by H_2_S. H_2_S can reduce the infarct size of cerebellar tissue and restore neurological function through mechanisms such as antioxidant, anti‐inflammatory, antiapoptotic, regulating autophagy, protecting mitochondrial function, and vasodilation and generation. By Figdraw. COX‐2, cytochrome oxidase subunit 2; γ‐GCS, γ‐glutamyl cysteine synthetase; iNOS, inducible nitric oxide synthase; mPTP, mitochondrial permeability transition pore; NF‐κB, nuclear factor‐kappa B; VEGF, vascular endothelial growth factor.

IRI of the brain is an important cause of ischemic stroke, mainly manifested by necrosis or softening of ischemic brain tissue and focal neuronal damage.^212^, ^213^ Stroke is the most common cause of disability in developed countries, and its high morbidity and mortality pose a great threat to the health of the whole population^214^, ^215^ Therefore, researchers need to find ways to detect and prevent ischemic strokes early. Fortunately, it has been found that appropriate concentrations of H_2_S have a neuroprotective effect on IRI in brain tissue.^216^, ^217^ Many experimental data suggest that the use of H_2_S donors to provide low concentrations of H_2_S can reduce infarct size and restore neurological function in brain tissue through mechanisms such as antioxidant, anti‐inflammatory, antiapoptotic, modulation of autophagy, protection of mitochondrial function, and vasodilation and angiogenesis.^218^, ^219^, ^220^, ^221^ The detailed mechanism is shown in Figure 5. In addition, it has been reported that H_2_S can also reduce infarct size and restore neurological function by modulating the expression of NMDA receptor expression levels, thereby activating the CREB pathway and improving neuronal cell survival.^222^, ^223^ However, another study showed that high concentrations of H_2_S inhibited COX activity in experimental mice, leading to brainstem toxicity and respiratory depression.^224^ These suggest that H_2_S plays a dual biological role in the brain.^225^, ^226^ Although the potential mechanisms of H_2_S in neuroprotection are poorly well understood and refined, there is no doubt that as a multitargeted neuromodulator, H_2_S has a very bright application in treating ischemic stroke.

### 4.4 H_2_S and the digestive system

#### 4.4.1 Nonalcoholic fatty liver disease

Nonalcoholic fatty liver disease (NAFLD) is the most common liver disease, which is mainly related to the abnormal accumulation of triglycerides in hepatocytes.^227^ CSE, CBS, and 3‐MST are all expressed in the liver, which contribute to the endogenous production of H_2_S in the liver, and the disruption of the metabolism of H_2_S may be related to the development of liver disease. In mice given a high‐fat diet (HFD), researchers found that the HFD induced lipid accumulation in the liver and disrupted normal liver structure compared with the normal diet group. However, the H_2_S‐treated group reversed this phenomenon, improving liver structure, reducing triglyceride and total cholesterol accumulation, decreasing fatty acid synthase expression, and increasing SOD and glutathione peroxidase activity. This experiment showed that H_2_S alleviated fatty liver by improving lipid metabolism and increasing antioxidant capacity.^228^ It has also been shown that plasma H_2_S levels are positively correlated with HDL cholesterol and negatively correlated with LDL cholesterol.^229^ Regarding the mechanism by which H_2_S affects NAFLD, it is still unclear. Still, a previous study by our group found that H_2_S reduced apoptosis and promoted autophagy through the ROS‐mediated PI3K/AKT/ mTOR signaling pathway, thereby improving HFD‐induced NAFLD.^230^

#### 4.4.2 H_2_S and IBD

IBD is a chronic inflammatory condition of the gastrointestinal tract that includes two main types: ulcerative colitis and Crohn's disease.^231^ Inflammatory response and oxidative stress are the main features of IBD.^232^ When inflammation occurs, neutrophils infiltrate in large numbers, generating ROS, inducing the formation of neutrophil extracellular traps (NETs), and initiating chromatin polymerization.^233^, ^234^ It has been found that H_2_S donors can induce neutrophil apoptosis and prevent the formation of colitis NETs, thus exerting an anti‐inflammatory effect.^235^ In addition, another study found that H_2_S donor treatment reduced the expression level of the oxidative stress marker 3‐NT and increased the expression of the antioxidants GSSH and SOD in a chemically induced IBD model. This suggests that H_2_S may play a cytoprotective role in reducing oxidative stress in IBD by inducing the production of antioxidants.^236^ In colitis, H_2_S reduces neutrophil infiltration and decreases the expression of several proinflammatory factors, such as IL‐6, IL‐1β, TNF‐α, and NO.^237^, ^238^ In addition to acting alone, H_2_S can also act as an anti‐inflammatory in conjunction with NO or CO.^8^, ^239^ Although most studies have demonstrated that H_2_S exerts an anti‐inflammatory effect, some experiments have found that higher concentrations of H_2_S increase cytotoxic effects and exacerbate the effects of IBD.^64^, ^240^ Therefore, the relationship between H_2_S and IBD is complicated, and the effect on IBD is related to the concentration of H_2_S, the type of donor, and the release rate, for which we should carry out a more in‐depth and accurate study.

### 4.5 H_2_S and cancer

Cellular metabolic reprogramming is one of the hallmarks of cancer and an inevitable requirement for the survival and proliferation of cancer cells, which helps them to adapt more rapidly to their environment and gain energy.^241^ In recent years, protein S‐persulfidation (P‐SSH) has gradually entered the field of vision of researchers, expanding a new way of thinking in cancer research. H_2_S is the most widely studied sulfur‐containing gaseous transmitter, and its role in cancer is known to be a double‐edged sword, with low concentrations of H_2_S promoting the development of cancer and high concentrations having the opposite effect.^242^ So there are two options for using H_2_S to treat cancer, that is, inhibition of endogenous H_2_S synthesis or exogenous donor supplementation of H_2_S.^19^ Currently, the molecular mechanisms of H_2_S in cancer are uncertain, but one more recognized mechanisms is the persulfidation modification of cysteine residues.^243^ A deeper understanding of P‐SSH's mechanism can help us better develop strategies to inhibit endogenous H_2_S and thus treat cancer.

Overcoming oxidative stress is particularly important for the development of cancer cells. Cysteine is a precursor for the synthesis of GSH and is also involved in the antioxidant response. Therefore, ensuring sufficient cysteine is also important for the development of cancer.^244^, ^245^ P‐SSH protects Cys residues from oxidative damage and reduces them to natural thiols under certain conditions, thus preserving protein function.^246^ In basal‐like breast cancer (BLCL), CBS overexpression has been reported to increase P‐SSH, which shields BLBC cells from damage caused by oxidative stress and promotes tumor cell proliferation and migration.^247^

Alterations in signaling pathways are also one of the characteristics of cancer, and it has been found that several key molecules of signaling pathways are already sulfur‐modified in different cancer types. For example, PTEN, an oncogenic factor of the PI3K/Akt signaling pathway, and protein tyrosine phosphatase 1B (PTPIB).^248^, ^249^ It has been reported that under the induction of H_2_S, PTEN undergoes P‐SSH modification at Cys‐71 or Cys‐124 and PTPIB at the Cys‐215 site. P‐SSH of these two molecules would inhibit themselves, activating the PI3K/Akt pathway and enhancing tumor cell proliferation.^248^, ^249^ Activation of the Akt pathway by exogenous H_2_S or donor administration has been reported in many tumor cell lines, for example, in colon cancer cells, thyroid cancer cells, and hepatocellular carcinoma cells.^250^, ^251^, ^252^

Epithelial–mesenchymal transition (EMT) is mandatory for tumors to undergo migration and invasion.^253^, ^254^ It has been found that silencing the endogenous H_2_S synthase CBS inhibits the EMT process in pancreatic ductal adenocarcinoma cells (PDAC).^255^ The experimental results showed that silencing CBS inhibited the migration and colony‐forming ability of PDAC. Meanwhile, cells silenced with CBS showed decreased expression of WNT5A, SANIL and decreased phosphorylation level of STAT3. STAT3 is an upstream molecule required for the Wnt signaling pathway, so the decrease in phosphorylation may further inhibit the Wnt pathway, thus hindering the EMT process in tumor cells.^256^ To dig deeper, the researchers did a metabolomic analysis. They found that silencing CBS leads to suppressed levels of protein persulfate, loss of oxidative protection of STAT3, and decreased levels of phosphorylation.^257^, ^258^

In summary, cancer cells can utilize P‐SSH to increase their ability to proliferate and migrate, are less susceptible to oxidative stress, and can better adapt to their environment.^259^ However, it is currently difficult to distinguish between H_2_S‐mediated P‐SSH and the organism's RSSH, making further mechanism exploration difficult. In addition to the endogenous H_2_S blocking therapies we mentioned above, providing exogenous H_2_S donors is also an effective option in suppressing cancer. GYY4137 inhibited the proliferation and migration of colorectal and hepatocellular carcinoma, and HA‐ADT inhibited the development of breast and esophageal squamous carcinoma.^260^, ^261^ As the most common H_2_S donor, the cancer‐inhibitory effects of high‐dose NaHS have been widely reported in such cancers as gastric cancer, melanoma, esophageal cancer, oral cavity cancer, thyroid cancer, prostate cancer, and neuroblastoma.^251^, ^262^, ^263^, ^264^, ^265^, ^266^

## 5 THERAPEUTIC POTENTIAL OF H_2_S AND H_2_S DONORS

### 5.1 H_2_S donors

#### 5.1.1 Sulfates

Sulfates are currently the most common H_2_S donors in biological research, such as sodium sulfide and NaHS, and have been shown to have protective effects on cells in disease states in multiple studies.^238^ Both sodium sulfide and NaHS exhibit a crystalline powder appearance, are easily soluble in water, and can provide H_2_S more directly. In early studies, Zhao et al. used NaHS aqueous solution to release H_2_S and found that it can reduce systemic arterial pressure, indicating that H_2_S has the characteristic of relaxing blood vessels.^47^ This has been verified in the research of Yoo et al.; in addition, they also found that the reduction of H_2_S donors will lead to the reduction of cardiac output, which will lead to the reduction of systemic arterial pressure.^267^ This phenomenon does not depend on the regulation of the CNS.^267^ In multiple studies, H_2_S released by exogenous donor NaHS can protect against organ damage, such as myocardial damage,^268^, ^269^ liver,^270^ brain,^271^ kidney,^269^ and so on. However, the chemical properties of sulfide salts are not stable, and the dosage and speed of H_2_S produced after direct dissolution in water are uncontrollable. The release of a large amount of H_2_S can cause a sudden drop in blood pressure, which has adverse effects on experimental animals.

#### 5.1.2 Lawesson reagent and their derivatives

Lawesson reagent (LR) is a common and readily available sulfur ion agent that can be an H_2_S releaser. The molecule of LR contains a quaternary ring with alternating sulfur and phosphorus atoms. Under high‐temperature conditions, the sulfur/phosphorus quaternary ring opens to form two unstable RCheicalbook‐PS2 (R‐PS2), which decompose and release H_2_S.^11^, ^272^ Compared with sulfide salts, LR releases H_2_S more slowly.^11^ However, LR's detailed release molecular dynamics still need to be clarified, and it lacks water solubility, so it has not been widely used as an H_2_S donor. GYY4137 is a new water‐soluble H_2_S donor synthesized based on LR reagent, which can slowly release H_2_S. Some studies have found that GYY4137 has the function of dilating blood vessels to resist hypertension.^273^ Not only that, it can also exert myocardial protection and prevent myocardial ischemia and reperfusion injury by inhibiting inflammation, reducing cell apoptosis, and reducing ROS production.^274^, ^275^ In addition to its myocardial protective effect, some scholars have found that in IRI, GYY4137 increases antioxidant activity by activating the Nrf2 signaling pathway, which can effectively alleviate renal injury.^276^ This protective effect has also been reported in testicular torsion and intestinal injury.^277^, ^278^ In addition, GYY4137 has been found to inhibit the proliferation and migration of colorectal and hepatocellular carcinomas, and it also relieves the pain caused by certain chemotherapeutic drugs such as paclitaxel.^279^, ^280^, ^281^ It can also relieve inflammation and reduce intestinal dysfunction when the intestinal barrier is compromised.^282^

#### 5.1.3 Sodium thiosulfate‐supplemented

Sodium thiosulfate is also a water‐soluble H_2_S donor with minimal side effects, and its chemical formula is Na_2_S_2_0_3_. STS is an antidote that has been approved by the US FDA and is currently mainly used in clinical practice to treat calcification reactions and toxic reactions (such as cisplatin poisoning, CO poisoning, cyanide poisoning, etc.).^283^, ^284^, ^285^ As mentioned earlier, in the metabolism of H_2_S, H_2_S can be oxidized to thiosulfate, and in turn, STS can also become a source of H_2_S. When the body is in a state of hypoxia, H_2_S can be regenerated in thiosulfate through 3‐MST and rhodase.^286^ In addition to releasing H_2_S, STS is also an effective antioxidant that has been proven to have strong protective effects in different organ injuries, such as acute liver injury,^287^ acute lung injury,^288^ brain injury caused by IRI,^289^ myocardial injury,^290^ kidney injury,^173^ and so on.

Studies have shown that STS may have anti‐inflammatory effects and protect vascular endothelial cells. H_2_S seems inhibit the NF‐κB signaling pathway and exerts anti‐inflammatory effects.^291^ Because of this cytoprotection on IRI, STS therapy has excellent potential in organ transplantation. The organ preservation solution added with STS is expected to become a simple, cheap, and safe new treatment strategy, which can reduce the transplant sequelae and improve the success rate.^292^

#### 5.1.4 Natural H_2_S donors and derivatives

Some natural foods can also serve as donors of H_2_S. Garlic is considered a good preventive food and has been found to have great medicinal research value.^293^, ^294^ Allicin is a metabolic active substance in garlic, which can be decomposed to produce diallyl polysulfides, such as diallyl sulfides, diallyl disulfides, and diallyl trisulfides (DATS).^295^ Moreover, these sulfides can react with GSH to produce H_2_S.^296^ However, due to the rapid release of H_2_S in water by DATS, some laboratories have utilized exploiting mesoporous silica nanoparticles (MSN) as a carrier to construct a new H_2_S release system (DATS‐MSN).^189^ DATS‐MSN can release H_2_S more slowly and controllably. In this study, compared with traditional H_2_S donors (NaHS, DATS, and GYY4137), DATS‐MSN showed better cardioprotective effects.^189^

#### 5.1.5 AP39

In addition, scientists have synthesized an H_2_S donor targeting mitochondria (AP39). AP39 can reduce intracellular oxidative stress and proinflammatory factor gene expression, maintain cell vitality, ensure mitochondrial energy and DNA integrity, and play an anti‐inflammatory and antioxidant cytoprotection.^297^ In mouse heart transplantation experiments, studies have found that adding AP39 to an organ preservation solution can significantly improve cell viability and reduce cold IRI and tissue fibrosis.^169^ AP39 can significantly reduce ROS production in mouse pancreatic transplantation experiments and improve pancreatic island function.^172^ These studies undoubtedly demonstrate the significant potential of AP39 in preventing and treating IRI in organ transplantation. As an H_2_S donor, in addition to protecting cells, AP39 can induce vascular relaxation by stimulating NO signaling and activating K_ATP_ channels.^298^ The development of AP39 shows that the development of specific target donors of H_2_S in subcellular organelles has great potential in future biological research.

### 5.2 Potential and future directions of H_2_S therapy

As we mentioned above, H_2_S therapy can be divided into endogenous and exogenous; endogenous inhibition of H_2_S production can be achieved not only by using H_2_S synthase inhibitors A0AA, PAG, or L‐ASP but also by using targeted drugs to prevent persulfuration modification, which can contribute to the oxidative damage of the cancer cells and make them more susceptible to death.^299^, ^300^, ^301^, ^302^, ^303^, ^304^, ^305^, ^306^, ^307^ For exogenous H_2_S therapy, the manner of H_2_S delivery is important. At present, the main methods of H_2_S delivery include direct inhalation and H_2_S donor. However, all these delivery methods have certain limitations. Gaseous H_2_S has a pungent odor and poor safety, and the concentration is difficult to control and cannot be delivered in a targeted manner. Hence, data stability could be improved and applied to human beings.^308^ Inorganic sulfates are currently the main H_2_S donors in biology and clinical trials, but they are still poorly targeted and require large amounts to be administered, which can lead to adverse reactions.^309^ Although recent studies have identified many small molecule H_2_S donors, they still perform poorly regarding stability, solubility, targeting, and half‐life.^309^ Therefore, designing safe, controllable, and stable targeting methods for H_2_S delivery is particularly important. Nowadays, the main way to constitute an H_2_S delivery system is to combine H_2_S donors with polymers by covalent linkage and physical entrapment. Most of these polymers, such as micelles,^310^, ^311^ hydrogels,^312^ liposomes,^313^ and nanoparticles, have good biocompatibility. The delivery systems optimize the therapeutic efficacy with higher stability and bioavailability compared with small molecule H_2_S donors.

To further improve the efficacy of H_2_S therapy, H_2_S delivery systems with intelligent properties have been carried out in recent years. Intelligent delivery systems allow for a stable and controlled release of H_2_S by specifically targeting and responding to the pathological microenvironment and external stimuli. In addition, the system monitors the release of H_2_S through the use of materials with imaging capability.^308^ Zhao et al.^314^, ^315^ designed an N‐mercapto (N‐SH)‐based H_2_S delivery system that not only releases H_2_S in a controlled manner, but also displays potent myocardial protection in a mouse model of myocardial IRI. Takatani‐Nakase et al.^316^ found that ADT nano micelle could release H_2_S in an in vitro ischemia model and were more effective in blocking cardiomyocyte apoptosis than NaHS and ADT‐OH. Sun et al^317^. constructed a targetable H_2_S delivery system (DATS@MION–PEG–LF) based on mesoporous iron oxide nanoparticles (MION). The system is mainly composed of MION, DATS, polyethylene glycol (PEG), and lactoferrin (LF). To prolong circulation time, the researchers modified PEG into MIONs, while LF helps the system target the brain through the blood–brain barrier. The study found that DATS@MION–PEG–LF can be taken up by neuronal cells and cardiomyocytes, protecting the brain and heart by exerting antiapoptotic and oxidative effects in IRI. More notably, this process can also be visualized by MRI.^317^ Hsieh et al. prepared a nanoparticle system loaded with DATS (DATS@MPs).^318^ Compared with free DATS, DATS@MPs can release H_2_S more slowly and for a more extended period. In a mouse model of hind limb ischemia, DATS@MPs can limit apoptosis, protect cells, and promote angiogenesis, which is beneficial for hind limb recovery.^318^

In addition to its cytoprotective role in IRI, researchers have developed smart delivery systems for H_2_S that have therapeutic effects in other diseases such as IBD, angiogenesis, disc degeneration, and rheumatoid arthritis.^319^, ^320^, ^321^, ^322^, ^323^ He et al. used bovine serum albumin (BSA) as a template to design MnS@BSA. MnS@BSA dissociates in a weakly acidic environment, releasing H_2_S and Mn^2+^. H_2_S can be used for gas therapy for cancer, and Mn^2+^ can not only be used for MRI, but it produces H_2_O_2_ that can have a synergistic anticancer effect with H_2_S.^324^ In exception to this, other smart delivery systems with anticancer effects have also been reported in large numbers.^310^, ^313^, ^325^, ^326^, ^327^, ^328^

We summarize the progress of the H_2_S smart delivery system in preclinical studies (summarized in Table 1) with the aim of more clearly and unambiguously describing the therapeutic potential of H_2_S and donors.

**TABLE 1:** Summary of advances in H_2_S donors and smart delivery systems in preclinical studies.

Therapeutic potential	H_2_S donors/polymeric carriers	Experimental models	Proposed mechanisms	References
Myocardial protection	NaHS	Neonatal cardiomyocytes IR model(rat)	Decrease of ROS level via downregulation of NF‐κB and JAK2/STAT3 pathways	188
	NaHS	Infarction model(mice)	Upregulation of Bcl‐2, demoted expression of Bax, IL‐1β, and Caspase 3	191
	NaHS	Myocardial I/R model(mice)	Antioxidant and antiapoptotic	190
	NaHS	Neonatal cardiomyocytes IR model(rat)	Inhibition of autophagy through regulation of the PI3K/SGK1/GSK3β signaling pathway	193, 194
	NaHS	Myocardial I/R model(mice)	Mitochondrial K_ATP_ channel opening	329
	GYY4137	SICM model(mice)	Inhibition of inflammatory response and reduction of ROS generation via NLRP3 pathway	274
	STS	Myocardial I/R model(rat)	Antioxidant and antiapoptotic	290
	DATS‐MSN	Myocardial I/R model and cardiomyocytes(rat)	Antioxidant, anti‐inflammatory, and antiapoptotic	189
	AP39	Heart transplant model(mice)	Blocked prolonged cold IRI and reduced tissue damage and fibrosis	169
	ADT micelle	Ischemic cardiomyocytes(mice)	Antiapoptotic	316
	DATS@MION–PEG–LF	Hypoxia/reoxygenation model and CA/CPR model(mice)	Antioxidant, anti‐inflammatory and antiapoptotic	317
Antiatherogenic	NaHS	Apolipoprotein‐E K.O. model (mice)	Inhibition of ICAM‐1 and TNF‐α signaling pathway	330, 331
	APA/SATO	HUVEC	Improved cell proliferation and migration	332, 333
Relief of pulmonary arterial	ACS14 MSs	PAH model(rat), HPAEC	Suppressed NF‐κB–Snail pathway	334
Relief of ND	GYY4137	3xTg‐AD mice	Prevented hyperphosphorylation of Tau by sulfhydrating GSK3β	206
	NaHS	PD mice	Increased SIRT1 expression and sulfhydration	210
Relief of ischemic stroke	NaHS	Cerebral I/R model(rat)	Improved SOD activity, removed oxygen free radicals, inhibited lipid peroxidation, and downregulated the expression of HSP70	219
Relief of NAFLD	GYY4137	NAFLD model(mice)	Inhibition of hepatic ER stress through the SIRT1/FoxO1/PCSK9 pathway	335
Relief of IBD	Lawesson's reagent and SASP	Colitis model(rat)	Inhibition of NETs formation to exert anti‐inflammatory effects	235
	CAP‐w‐FC	Colitis model(rat)	Anti‐inflammatory	319
Anticancer	NaHS	A549 cell	Regulation of the TGF‐β1/Smad2/Smad3 pathway inhibits EMT protein and migratory capacity	264
	GYY4137	Colon cancer cell	Downregulation of CD44 inhibits tumor cell proliferation and metastasis	280
	GYY4137	Subcutaneous HepG2 xenograft model(mice)	Blocked STAT3 pathway	281
	mPEG‐SSS‐cholesteryl (T)	Coculture model of fibroblasts and breast cancer cells	Reduced ROS levels	325
	SATO‐functionalized micelle	Colon cancer cell	Targeted cell toxicity	310
	MnS@BSA	4T1 cell, xenograft model(mice)	Inhibition of tumor growth	324
	FeS@BSA	Huh7 cell, xenograft model(mice)	Inhibition of tumor growth	328

However, smart H_2_S delivery systems are still in their infancy, and there is still a long way to go from the lab to the clinic. First, the pharmacological research of H_2_S needs to be more profound, and the detailed molecular mechanism and targets of its action in vivo need to be clarified. Second, it is not easy to monitor the location and concentration of H_2_S accurately using current commonly used technologies. Therefore, H_2_S delivery systems rely on more in‐depth biological research. However, we believe that through innovation and improvement in all aspects, the intelligent H_2_S delivery system will eventually unleash its great potential.

## 6 DISCUSSION

There is increasing evidence that reasonable concentrations of H_2_S can have protective effects in physiological and pathological conditions, possibly through antiapoptosis, regulation of autophagy, and inhibition of oxidative stress and inflammation. The growing understanding of the important biological effects of H_2_S, such as vasodilatory, cytoprotective antioxidant, and anti‐inflammatory effects, as well as its signaling pathway mechanisms, has facilitated the translation of the highly promising cytoprotective functions of H_2_S into more viable clinical therapeutic modalities.

Key to this is the effective design of H_2_S donors to deliver the desired therapeutic effects. As discussed earlier, designing stable, controlled H_2_S donors that maintain a stable and slow release of H_2_S over time is preferable for clinical applications, and much of the physiological utility of H_2_S is derived from its redox properties. The uncontrolled and rapid release of H_2_S donors rapidly alters the redox state of cells, which has a much greater impact on cells than its beneficial physiological functions. With rapidly increasing H_2_S concentrations, the distribution of each different oxidation state sulfide is vastly different from the normal physiological state, yet each sulfide has its unique physiological properties.

The volatility of H_2_S and its rapid metabolism makes the development of H_2_S donors uniquely challenging compared with the development of other small molecule donors, which are highly volatile and are always in a dynamic, volatile‐soluble equilibrium. In addition, many of the current H_2_S donors are polysulfides, both the donor itself and the by‐products of H_2_S fraction production, so it is often difficult to distinguish whether the physiological effects of such donors are derived from H_2_S or other polysulfides. Another difficulty in H_2_S research is how to quantify the range of endogenous H_2_S concentrations during human circulation and the changes in H_2_S concentrations during treatment. This is mainly due to the reactive chemical nature of H_2_S and the complex environment of sulfides in vivo. The inability to accurately monitor H_2_S concentrations in the circulatory system or target organs will make it difficult to assess the exact relationship between H_2_S and physiological effects. Therefore, it is important to develop methods to quantitatively detect H_2_S concentrations in vivo for H_2_S research.

In conclusion, although sulfide generators are not new drugs to date, there is precedent for reducing metabolism, thus providing protection against IRI in humans. For example, hypothermia therapy has been shown to be beneficial for outcomes in a variety of situations, including out‐of‐hospital cardiac arrest and during myocardial revascularization. Although many issues still need to be addressed, these critical issues must be resolved to move into clinical treatment. However, future multidisciplinary collaborations involving nanomaterials, chemistry, pharmaceutical, and biological disciplines may finally offer a possibility for H_2_S therapy, and we look forward to seeing more exciting studies in this area.

## AUTHOR CONTRIBUTIONS

*Dong‐Dong Wu, Zhi‐Guang Ren, and Xin‐Ying Ji*: conceived and supervised the study. *Yu‐Qing Jin, Hang Yuan, Ya‐Fang Liu, Yi‐Wen Zhu, Yan Wang, Xiao‐Yi Liang, and Wei Gao* drafted the manuscript and prepared the figures. All authors read and approved the final manuscript.

## CONFLICT OF INTEREST STATEMENT

The authors declare that they have no conflict of interest.

## ETHICS STATEMENT AND CONSENT TO PARTICIPATE

Not applicable.

## Data Availability

Not applicable.
